# Supplementary material for: Efficient stabilization of cyanonaphthalene by fast radiative cooling and implications for the resilience of small PAHs in interstellar clouds
Source: Nat Commun. 2023 Jan 24;14:395. doi: 10.1038/s41467-023-36092-0 (PMC9873784; doi:10.1038/s41467-023-36092-0)
Supplement: Supplementary file 1 — Supplementary Information: Efficient stabilization of cyanonaphthalene by fast radiative cooling and implications for the resilience of small PAHs in interstellar clouds [file 41467_2023_36092_MOESM1_ESM.pdf]

Supplementary Information: Efficient stabilization of cyanonaphthalene by fast radiative cooling and implications for the resilience of small PAHs in interstellar clouds

Mark H. Stockett[1*], James N. Bull[2], Henrik Cederquist[1], Suvasthika Indrajith[1], MingChao Ji[1], José E. Navarro Navarrete[1], Henning T. Schmidt[1], Henning Zettergren[1] & Boxing Zhu[1]

[1]Department of Physics, Stockholm University, Stockholm, Sweden
[2]School of Chemistry, University of East Anglia, Norwich, United Kingdom
*Mark.Stockett@fysik.su.se

Supplementary Methods

The detector efficiency $\eta_{det} = 0.34(3)$ was determined by equating the absolute dissociation rate in the single-pass measurement to that during the first revolution of ions in the stored-beam measurement. Both rates correspond to ions which decay $t_{init} = 120 - 124$ μs after formation. For the single-pass measurement,

$$\Gamma(t_{init}) = \frac{1}{N(t_{init})}\frac{dN}{dt} = \frac{q_e f_{rev}}{I_{sp}G}\frac{n_{\mathrm{HCN}}}{\eta_{det}\Delta t_{sp}}, \quad (1)$$

where N is the number of ions in the observation arm, $G = L_{SS}/C$ is the fraction of the ring seen by the detector, I_{sp} is the measured beam current after a single pass, q_e is the unit charge, $f_{rev} = 23.87$ kHz is the revolution frequency for 1-CNN$^+$ at 34 keV, and n_{HCN} is the number of neutral HCN fragments detected during the time $\Delta t_{sp} = 396$ s the camera was exposed.

For the stored-beam measurement,

$$\Gamma(t_{init}) = \frac{1}{\eta_{det}GN(t_{init})}R(t_{init}). \quad (2)$$

Equating Eq. 1 with Eq. 2, the detection efficiency cancels. However, it re-enters calculation when determining the initial number of ions $N(t_{init})$ in the stored-beam measurement (Eq. 2). In that experiment, the number of ions remaining in the ring is measured at the end of each cycle at $t = t_{fin}$ by dumping the beam into the Faraday cup. The number of ions at the beginning of the cycle is found from

$$N(t_{init}) = N(t_{fin}) + \int_{t_{init}}^{t_{fin}} \frac{dN}{dt}dt = \frac{I_{fin}}{f_{rev}q_e} + \frac{1}{\eta_{det}GY_{\mathrm{HCN}}}\int R(t)dt, \quad (3)$$

where I_{fin} is the measured beam current, averaged over the number of storage cycles, and $Y_{\mathrm{HCN}} = 0.70(5)$ is the branching fraction for HCN-loss estimated from published breakdown curves[1]. The other main dissociation product, atomic hydrogen, has a negligible detection efficieny due to its low kinetic energy[2]. Setting equal the rates from Eqs. 1 and 2 with Eq. 3 inserted, we find

$$\eta_{det} = \frac{\zeta_{sp} f_{rev}\int R(t)dt}{GY_{\mathrm{HCN}}(R(t_{init}) - \zeta_{sp}I_{fin}/q_e)}, \quad (4)$$

where $\zeta_{sp} = n_{\mathrm{HCN}}q_e/I_{sp}\Delta t_{sp}$.

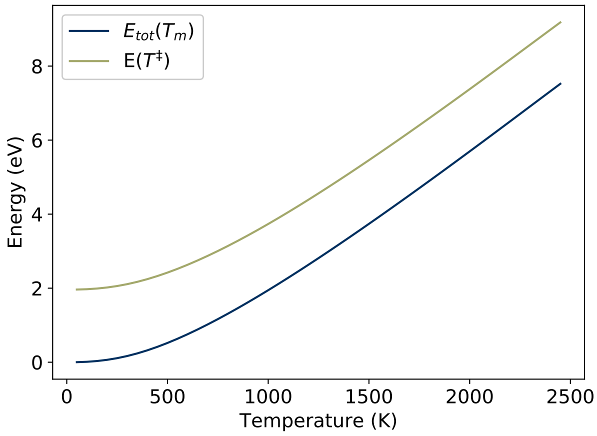

Supplementary Fig. 1 Caloric curves $E_{tot}(T_m)$ (Eq. 5), calculated from vibrational level densities, and $E(T^‡)$ (Eq. 6), including the finite heat bath correction.

Supplementary Figures

Figure 1 gives the caloric curve for 1-CNN$^+$. The total vibrational energy $E_{tot}(T_m)$ is computed assuming Boltzmann statistics:

$$E_{tot}(T_m) = \frac{\int E'\rho(E')e^{-E'/k_BT_m}dE'}{\int \rho(E')e^{-E'/k_BT_m}dE'} \quad (5)$$

where T_m is the microcanonical temperature. The vibrational energy E corresponding to the transition state temperature $T^‡$ is computed using the second order finite heat bath correction[3]:

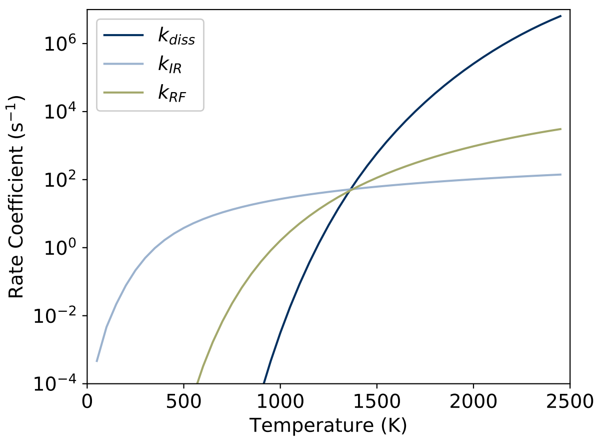

Supplementary Fig. 2 Thermally averaged rate coefficients (Eq. 7).

$$E = E_{tot}(T^{\ddagger}) + \frac{E_a}{2} + \frac{E_a^2}{12(E_{tot}(T^{\ddagger}) + E_a/2)}. \quad (6)$$

Figure 2 gives the thermal averages of the microcanonical rate coefficients from Fig. 4A in the main text, *i.e.*:

$$k(T_m) = \frac{\int k(E')\rho(E')e^{-E'/k_B T_m} dE'}{\int \rho(E')e^{-E'/k_B T_m} dE'}. \quad (7)$$

Supplementary Note 1: Competing Dissociation Channels

We expect the measured count rate $R(t)$ to be dominated by dissociation of 1-CNN$^+$ yielding HCN molecules according to:

$$C_{10}H_7CN^+ \longrightarrow C_{10}H_6{}^+ + HCN. \quad (8)$$

Two other dissociation channels were identified by West *et al.* in their study of dissociative ionization of cyano-substituted PAHs[1]:

$$C_{10}H_7CN^+ \longrightarrow C_{10}H_6CN^+ + H \quad (9)$$

and

$$C_{10}H_7CN^+ \longrightarrow C_8H_5CN^+ + C_2H_2. \quad (10)$$

In this section we present an alternative kinetic model which includes these channels, which are neglected in the model presented in the main text. For Eq. 9, we estimate the dissociation rate coefficient from the branching ratio for this channel from West *et al.* and the presently determined rate coefficient for HCN-loss (Eq. 8), which has a similar appearance energy according to West et al. Following the finding that cyano-substitution has little impact on the rate coefficient for C_2H_2-loss, we adopt a rate coefficient for Eq. 10 equal to that for unsubstituted naphthalene given by West et al.[6] (another one!). The rate coefficients are parameterized according to[7]:

Fragment	A^{diss}_{1000K} 10^{13} s^{-1}	E_a eV	Source
HCN	2	3.16(4)	Present
H	3	3.26(3)	West *et al.*[1]
C_2H_2	30	4.12(5)	West *et al.*[6]

Supplementary Table 1: Parameters for dissociation rate coefficients used in the alternative kinetic model.

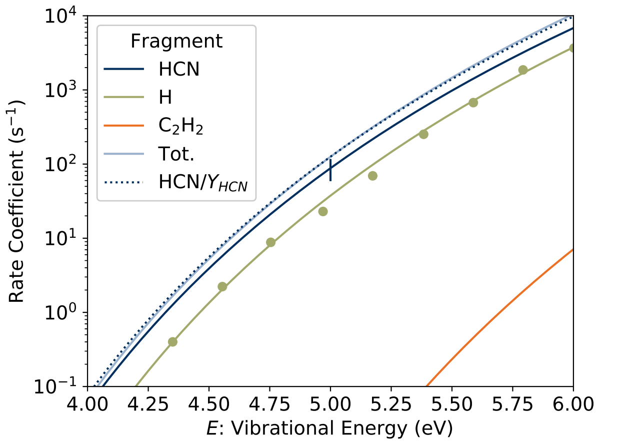

Supplementary Fig. 3 Estimated rate coefficients for competing dissociation channels. Symbols are the product of the presently determined rate coefficient for HCN-loss and the branching fractions for H-loss from West *et al.*[1]; the solid green line is a fit of Eq. 11 to these points. The rate coefficient for C_2H_2-loss is that adapted from that for naphthalene taken from West *et al.*[6] 'Tot.' is the sum of the three fragmentation channels, which is nearly indistinguishable from the approximate value of $k^{\mathrm{HCN}}_{diss}/Y_{\mathrm{HCN}}$ used in the main text. The uncertainty in the rate coefficients is indicated at the single point at 5 eV.

$$k_{diss}(E) = A^{diss}_{1000K}\frac{\rho(E - E_a)}{\rho(E)}, \quad (11)$$

with the parameters given in Table 1, and are plotted in Fig. 3.

The products of the competetive H-loss channel are detected with very low efficieny due to their low kinetic energy[2]. The C_2H_2-loss channel has a much higher appearance energy and is not expected to significantly tribute to $R(t)$. Fig. 4 shows a simulation of $R(t)$ based on an alternative master equation model including these three dissociation channels. This simulation uses the same radiative cooling rate coefficients described in the main text and is initialized to the best fitting initial temperature of 1970 K. The decay rates are scaled by their detection efficiencies, using $\eta_{det} = 0.34$ for HCN and C_2H_2 fragments and 0.02 for H[2]. Assuming these efficiencies, HCN-loss accounts for no less than 97% of the total simulated count rate.

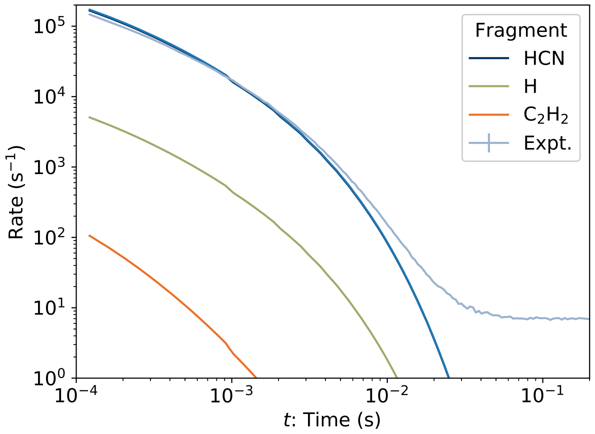

Supplementary Fig. 4 Simulated count rate for alternative kinetic model.

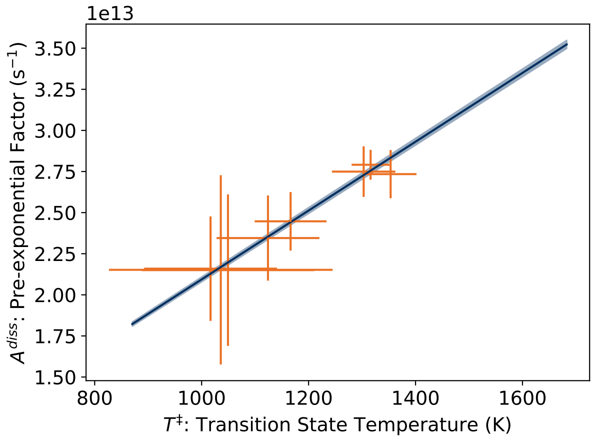

Supplementary Fig. 5 Fit to Eyering equation (Eq. 13) to obtain $\Delta S_{1000\text{K}}$. The error bars are the standard deviations of the fit parameter estimates.

Supplementary Note 2: Determination of $\Delta S^{\ddagger}$

In the main text, we determine E_a from a simultaneous fit to the KER distributions recorded up to 20 ms after formation. Numerically, this procedure is facilitated by eliminating the parameter $\Delta S^{\ddagger}$ from the model, which is justified in the present case by the negligible reverse barrier energy ΔE. As this is not generally the case, we present here a method for determining $\Delta S^{\ddagger}$ from individually fit KER distributions.

Given the parameters parameters $T^{\ddagger}$, ΔE, and $\hbar\omega$ extracted from each KER distribution, the value of pre-exponential factor A^{diss} is given by[4]:

$$A^{diss} = \frac{1}{h}\int_0^{E-E_a} \frac{e^{\beta}}{e^{\beta}+1} e^{-(\epsilon-\Delta E)/k_B T^{\ddagger}} d\epsilon$$

$$\beta = 4\pi\frac{\Delta E}{\hbar\omega}\left(\sqrt{1+\frac{\epsilon}{\Delta E}}+1\right). \quad (12)$$

As shown in Fig. 5, we find a linear relationship between $T^{\ddagger}$ and A^{diss}, which we fit to the Eyring equation:

$$A^{diss} = \frac{k_B T^{\ddagger}}{h} e^{1+\frac{\Delta S^{\ddagger}}{N_A k_B}} \quad (13)$$

where N_A is Avogadro's number[5]. We obtain a formation entropy $\Delta S^{\ddagger} = -8.27(6)$ $\text{JK}^{-1}\text{mol}^{-1}$, or $1 + \frac{\Delta S^{\ddagger}}{N_A k_B} = 5(7) \times 10^{-3}$. That this is close to zero, and the large covariance apparent between the parameters plotted in Fig. 5, justify eliminating $\Delta S^{\ddagger}$ from the model.

Supplementary References

[1] West, B. J., Lesniak, L. & Mayer, P. M. Why do large ionized Polycyclic Aromatic Hydrocarbons not lose C_2H_2? *J. Phys. Chem. A* **123**, 3569–3574 (2019).

[2] Stockett, M. H. *et al.* Unimolecular fragmentation and radiative cooling of isolated PAH ions: A quantitative study. *J. Chem. Phys.* **153**, 154303 (2020).

[3] Andersen, J., Bonderup, E. & Hansen, K. On the concept of temperature for a small isolated system. *J. Chem. Phys.* **114**, 6518–6525 (2001).

[4] Hansen, K. Tunneling and reflection in unimolecular reaction kinetic energy release distributions. *Chem. Phys. Lett.* **693**, 66–71 (2018).

[5] Leyh, B. Ion dissociation kinetics in mass spectrometry. In Lindon, J. C. (ed.) *Encyclopedia of Spectroscopy and Spectrometry (Second Edition)*, 1127–1134 (Academic Press, Oxford, 1999), second edn.

[6] West, B. *et al.* On the dissociation of the naphthalene radical cation: New ipepico and tandem mass spectrometry results. *J. Phys. Chem. A* **116**, 10999–11007 (2012).

[7] Boissel, P., de Parseval, P., Marty, P. & Lefvre, G. Fragmentation of isolated ions by multiple photon absorption: A quantitative study. *J. Chem. Phys.* **106**, 4973–4984 (1997).
